# Close-up observations on the spawning behavior of a captive Japanese flying squid (*Todarodes pacificus*)

**DOI:** 10.1038/s41598-019-56071-0

**Published:** 2019-12-24

**Authors:** Jun Yamamoto, Kohsuke Adachi, John R. Bower, Hajime Matsui, Mitsuhiro Nakaya, Ryusei Ohtani, Pandey Puneeta, Satoshi Suzuki, Shun Tokioka, Dharmamony Vijai, Takashi Yanagimoto, Hae-Kyun Yoo

**Affiliations:** 1 Field Science Center for Northern Biosphere, Hokkaido University, 3-1-1 Minato, Hakodate, Hokkaido, 041-8611 Japan; 2 Faculty of Agriculture and Marine Science, Kochi University, Mononobe-otsu 200, Nankoku, Kochi, 783-8502 Japan; 3 Faculty of Fisheries Sciences, Hokkaido University, 3-1-1 Minato, Hakodate, Hokkaido, 041-8611 Japan; 4 Graduate School of Fisheries Sciences, Hokkaido University, 3-1-1 Minato, Hakodate, Hokkaido, 041-8611 Japan; 5 Tohoku National Fisheries Research Institute, Japan Fisheries Research and Education Agency, 25-259 Shimomekurakubo, Samemachi, Hachinohe, Aomori, 031-0841 Japan; 6 Graduate School of Environmental Sciences, Hokkaido University, 3-1-1 Minato, Hakodate, Hokkaido, 041-8611 Japan; 7 Shizuoka Prefectural Research Institute of Fishery, Izu Branch, 251-1 Shirahama, Shimoda, Shizuoka, 451-0012 Japan; 8 National Centre for Biological Sciences, Tata Institute of Fundamental Research, Bellary Road, Bangalore, 560065 Karnataka, India; 9 National Research Institute of Fisheries Science, Japan Fisheries Research and Education Agency, 2-12-4 Fukuura, Yokohama, Kanagawa 236-8648 Japan; 10 East Sea Fisheries Research Institute, National Institute of Fisheries Science, 1194, Haean-ro, Yeongok-myeon, Gangneung, Gangwon, 25435 Republic of Korea

**Keywords:** Behavioural ecology, Ecology, Zoology, Animal behaviour

## Abstract

The spawning behavior of a Japanese flying squid (*Todarodes pacificus*) is described based on up-close observation of a captive female. The squid was first transferred from a 10-ton tank to a polystyrene plastic box containing 45 liters of seawater. About one hour later, the mantle-contraction rate increased rapidly, followed by a brief convulsion of the mantle and arms and a whitening of the body. The mantle contractions become shallow and rapid, and several seconds later, semitransparent jelly presumably from the nidamental glands emerged from the funnel and passed between the ventral pair of arms. Approximately 90 seconds after the egg mass first emerged, the female began ejecting oocytes through the funnel into the egg mass using rapid, powerful mantle contractions. Soon after the oocytes were ejected, translucent strands (presumably sperm) emanated from the buccal membrane. The female continued to eject oocytes for approximately two minutes, after which the mantle convulsed, and the mantle-contraction rate decreased slowly for about one minute until the contractions stopped. The squid died soon afterwards.

## Introduction

Cephalopod behavior is complex and has intrigued humans for centuries. Some of the most diverse behaviors are related to reproduction^1^. Our knowledge of cephalopod reproduction has grown much during the past two decades, but most of the research has focused on nearshore species, such as cuttlefishes and myopsid squids (reviewed by Hanlon and Messenger^1^), and idiosepiids (e.g.,^2,3^); little is known about the oegopsid squids, which occur further offshore.

The Japanese flying squid (*Todarodes pacificus*) is a nerito-oceanic, ommastrephid species that occurs widely in continental shelf and slope waters in the northwest Pacific Ocean^4^. It is the target of the largest squid fishery in Japan and is usually one of the three largest cephalopod fisheries in the world^5,6^. The species undertakes a long-distance, southerly migration and spawns mainly during autumn and winter in the southwest Sea of Japan and northern East China Sea^7^.

**Figure 1:**
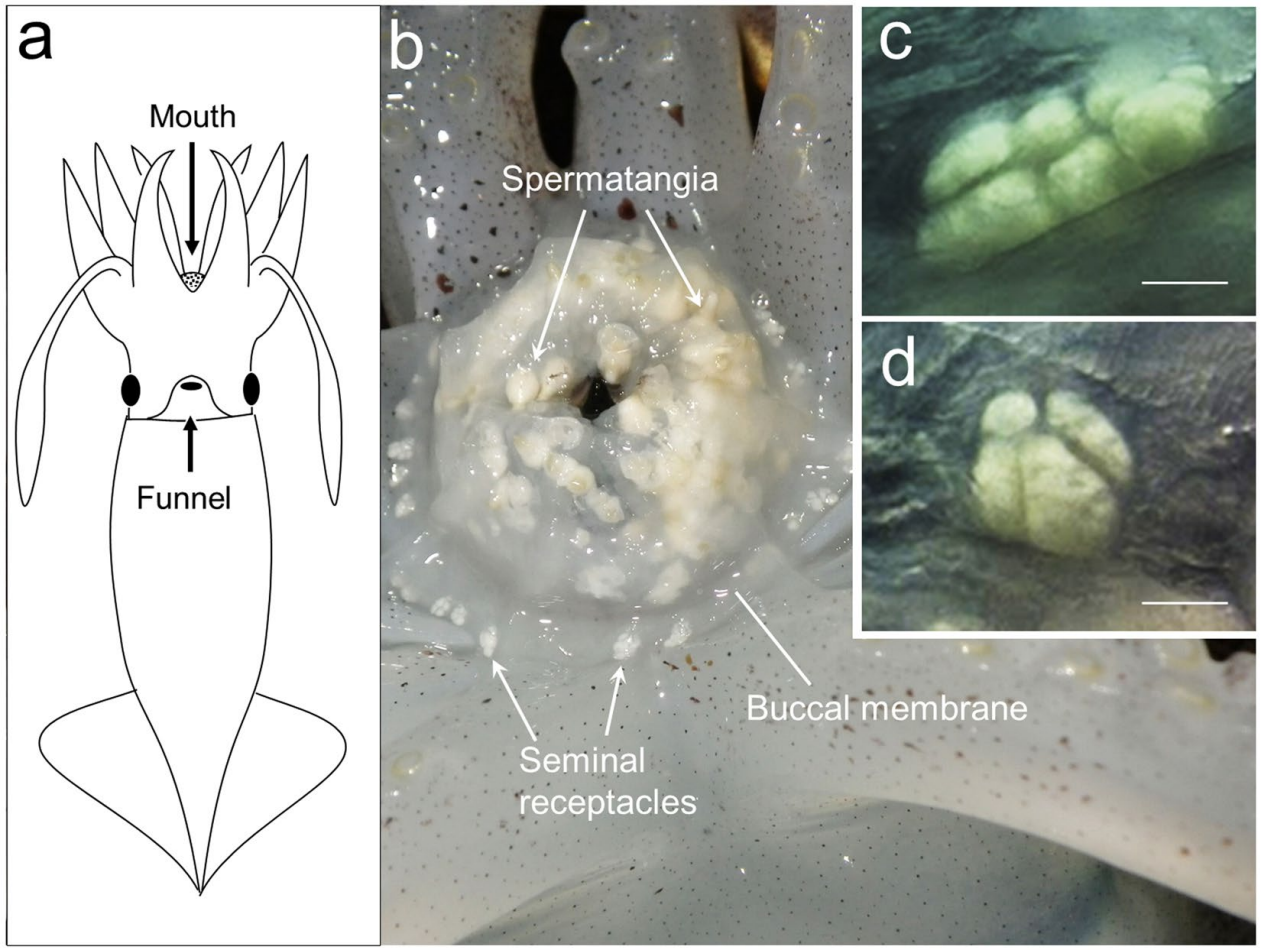
(**a**) Ventral view of a Japanese flying squid (*Todarodes pacificus*). (**b**) Mouth area of a copulated female before spawning. (**c**,**d**) Seminal receptacles, which usually comprise several sperm chambers. Bar = 0.5 mm.

The reproductive behavior of *T. pacificus* has not been observed *in situ*, but has been described based on laboratory studies^8–10^. During mating, a male deposits spermatophores onto a female’s mouth (Fig. 1). The spermatophores release a sperm packet (spermatangium), which attaches to a ring of fleshy tissue surrounding the mouth called the lips^11^. Sperm then move from the embedded spermatangium to seminal receptacles located on the buccal membrane. This movement has not been observed in *T. pacificus*, but Soeda^12^ observed spermatozoa emanating from the distal ends of spermatangia embedded on the lip and speculated that mated females might manipulate the buccal membrane to bring the spermatangia tips near the sperm receptacles. In the ommastrephid Humboldt squid (*Dosidicus gigas*), sperm released from the spermatangia actively migrate across the buccal membrane to the seminal receptacles, where they are stored alive until the female spawns^13^.

In the females, oocytes (defined in the present paper as unfertilized female gametocytes) develop in the ovary and are released and stored in the oviducts. During spawning, the oocytes pass through the oviducal glands and are presumably coated with oviducal-gland secretions, allowing them to hydrate and preparing them for later chorionic expansion. The oocytes then pass through the funnel and into a gradually ballooning egg mass surrounded by a layer of jelly from the nidamental glands^14^. Fertilization is thought to occur near the buccal area as the oocytes enter the egg mass, however this process is unclear. What triggers the release of sperm from the seminal receptacles is also not known.

Despite our growing knowledge about the spawning behavior of *T. pacificus*, many questions remain unanswered, particularly related to the release and fertilization of oocytes. The present study was conducted to better understand these processes.

## Results

### Pre-spawning

The squid swam calmly after it was transferred to the box. The mantle-contraction rate was 65-70 per minute. Mantle chromatophores continuously pulsated.

### Spawning

**Figure 2:**
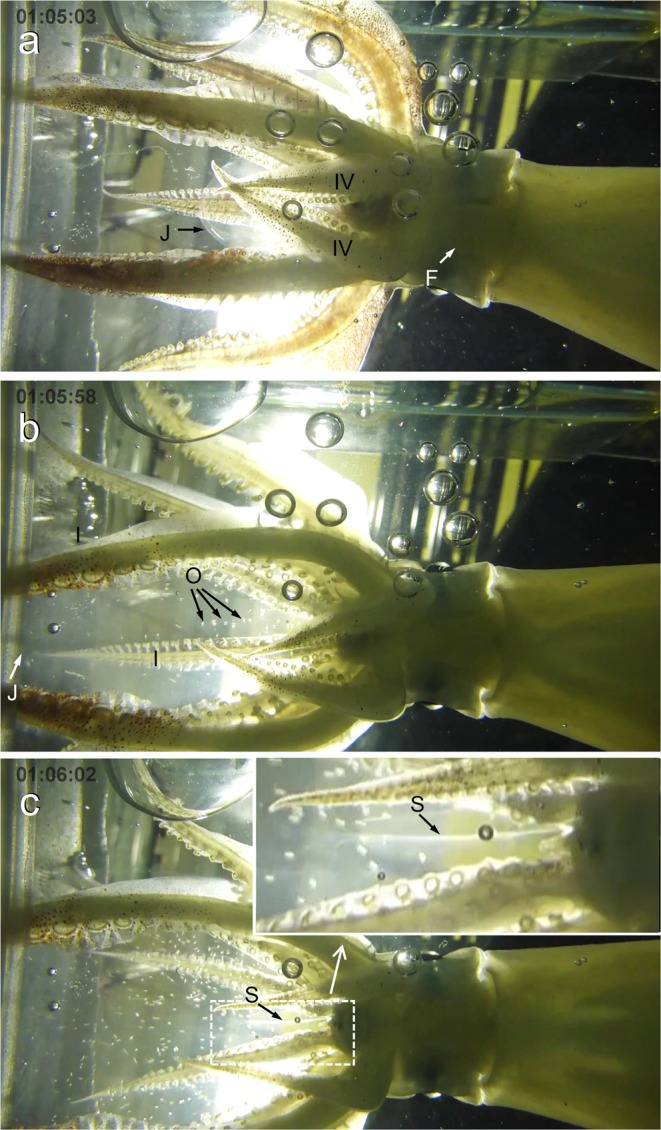
Stills from video of a spawning Japanese flying squid (*Todarodes pacificus*). (**a**) Squid slowly released cloudy but transparent jelly (J) through the funnel (F) and between the ventral pair of arms (arms IV). (**b**) When the jelly (J) reached near the tips of the dorsal pair of arms (arms I), the squid began to eject oocytes (O). (**c**) The oocytes were pumped rapidly and vigorously over the buccal membrane. Filament-like translucent strands of presumably sperm (S) issued from the buccal membrane. Elapsed time after transfer to the box is shown in the upper left.

Approximately 64 minutes after the transfer, the mantle-contraction rate increased rapidly, followed by a brief convulsion of the mantle and arms. Chromatophores on the mantle and arms retracted, causing most of the body to whiten. The mantle contractions become shallower and more rapid (approximately 83 per minute). Immediately after the convulsion, jelly began to emerge from the funnel and pass between the ventral pair of arms (arms IV) (Fig. 2a). At first, the ventral arm pair (arms IV) were straight, but as the jelly started to extrude, the arms became bent. The other arms (I-III) and tentacles remained fairly straight. The jelly was semitransparent and contained no oocytes. It continued to slowly emerge, reaching near the buccal membrane (mouth) after about ten seconds and extending to near the tips of the dorsal arm pair (arms I) after another 70 seconds (see Supplementary Information Video 1).

Approximately 90 seconds after the egg mass first emerged (Fig. 2b), the female began ejecting oocytes through the funnel into the egg mass using rapid, powerful mantle contractions (approximately 120-125 per minute). The egg mass expanded for about ten seconds, but then broke apart, presumably due to the limited space inside the box. The female continued to eject oocytes for approximately two minutes. During this time, the oocytes were pumped vigorously over the buccal membrane causing the lips to flutter. Filament-like translucent strands of presumably sperm were seen issuing from the buccal membrane (Fig. 2c) (see Supplementary Information Video 2).

Two minutes after the start of spawning, the mantle convulsed, and the mantle-contraction rate decreased slowly for about one minute until the mantle contractions stopped.

### Post-spawning

The squid stopped swimming after it ceased spawning and died within 20 minutes. Eggs were dispersed throughout the box. The number of eggs in the box were not counted, but visual estimates from video images indicate at least 2000 were spawned. Two days following the spawning, 98 eggs were randomly collected to estimate the fertilization rate. Approximately 29% were fertilized. Post-spawning dissection showed many oocytes remained in the oviducts, and spermatangia and seminal receptacles containing sperm remained near the mouth. The squid was classified as Stage V based the maturity scales of Lipinski and Underhill^15^.

## Discussion

Our results are the first up-close observations on spawning in an oegopsid squid and provide several important clues about how oocytes are released and fertilized.

Spawning began with the gradual extrusion through the funnel of jelly, presumably from the nidamental glands, which contained no oocytes. This is the first direct observation of this phenomenon and supports Hamabe’s^16^ suggestion that jelly extrusion precedes the discharge of oocytes. In his study, Hamabe kept mature females in barrels set on the seafloor and obtained egg masses and jelly. Based on these observations, he also suggested that the jelly adheres to substrate, which the results of our study and others do not support.

Soon after the squid began pumping oocytes into the egg mass, we observed what appeared to be sperm emanating from its buccal membrane. The ommastrephid squid *D. gigas* has circular muscles in its seminal-receptacle chambers, which may be used to extrude sperm^13^. A similar process could occur in *T. pacificus*, but what triggers this extrusion is not known. Durward *et al*.^17^ suggested that in the northern shortfin squid (*Illex illecebrosus*), an ommastrephid in which males implant spermatophores inside the female’s mantle cavity instead of around the mouth, sperm are activated by the nidamental gland jelly. In a similar manner, chemical factors in the jelly extruded by *T. pacificus* before the release of oocytes could trigger the release of sperm from the seminal receptacles on the buccal membrane. This is a topic that warrants further investigation.

We were not able to confirm how fertilization occurs, but a spawning female might actively release spermatozoa from her seminal receptacles and insert them into the gradually ballooning egg mass as it emerges from the funnel. The powerful flow of water we observed over the buccal membrane would disperse the spermatozoa into and throughout the egg mass. Another possibility is that spermatozoa are released and actively swim toward and into the egg mass after passing through the outer egg-mass layer created from the nidamental-gland jelly. The mechanism guiding this movement is unclear, but other cephalopods are known to use chemical cues to direct the movement of sperm – a process called “sperm chemotaxis”. In the common cuttlefish (*Sepia officinalis)*, sperm-attracting peptides direct sperm to the oocytes^18^. Similarly, chemoattractant peptides in eggs of the common octopus (*Octopus vulgaris)* increase sperm motility and induce chemotaxis^19^. And in *T. pacificus*, sperm have been shown to attract to CO_2_, which could be emitted from oocytes^20^. Fernández-Álvarez *et al*.^13^ showed that in *D. gigas*, sperm in spermatangia implanted in the buccal membrane actively migrate over the surface of buccal membrane to the seminal receptacles, which suggests the seminal receptacles also release a sperm-attracting substance. We suspect the oocytes and possibly the nidamental gland jelly in *T. pacificus* also play a chemotaxic role in directing sperm toward the oocytes, but further experimental evidence is needed to confirm this hypothesis.

Previous studies (e.g.,^10,16,21^) have suggested that fertilization occurs at or near the buccal membrane. Our observations on the vigorous pumping of oocytes past the buccal membrane and into the egg mass suggest the while sperm likely enter the egg mass near the buccal membrane, fertilization likely occurs deeper inside the egg mass. Each sperm released from the buccal area presumably has an equal chance or “fair raffle”^22^ in fertilizing a female’s ova. Cryptic female choice could occur if females are able to manipulate sperm through controlled release from the sperm receptacles^13^, but this also requires further study. Furthermore, the outer jelly of the egg mass likely facilitates fertilization by protecting sperm and oocytes, and reducing sperm dispersal into the environment.

The pre-spawning squid did not display signs of extreme stress. First, after transfer to the box, the squid swam calmly and maintained a steady mantle-contraction rate for about one hour. And when spawning commenced, the female’s ventral arms became bent similar to captive females that spawned in a large tank^10^, suggesting the female displayed normal spawning behavior. The observed pulsating chromatophores were also reported in previous studies in large tanks. Bower and Sakurai^8^ reported that about two days before spawning, the body chromatophores of pre-spawning females flash rapidly across the body surface in an incandescent pattern. This phenomenon was also reported by Puneeta *et al*.^10^. Conditions remained normal until the egg mass broke apart within the confined space. We believe the breaking apart of the egg mass was responsible for the low fertilization rate (29%) compared with previous studies by Bower and Sakurai^8^ and Puneeta *et al*.^10^, who both reported fertilization rates within unbroken egg masses spawned in captivity above 90%.

In conclusion, most of our knowledge of cephalopod reproduction has come from nearshore species that are accessible and that readily adapt to laboratory conditions. Oegopsid squids such as *T. pacificus* are more difficult to collect unharmed and rear long term for experiments like the one described in this paper. But to examine their reproductive behavior, laboratory studies are easier than studies at sea. Recent improvements in laboratory seawater systems and husbandry methods, as well as the creation of large experimental aquaria are creating more opportunities to study live cephalopod^1,23^, which we hope will help answer the many unresolved behavioral questions about reproduction in oceanic squids.

## Materials and Methods

### Squid

A total of 70 squid were collected on three dates during late August to mid-September 2016 by jigging aboard the Hokkaido University training vessel *Oshoro maru* in and around Tsugaru Strait (northern Japan) and transferred to an approximately 10-ton tank at the Hakodate Research Centre for Fisheries and Oceans, Japan (Hakodate). The squid were reared at 14-15 °C, during which they matured and mated. Mature females were visually identified by their enlarged oviducts (filled with amber-colored oocytes), nidamental glands, and oviducal glands, which became visible through the mantle while the squid swam in the tank.

### Experimental design

On September 19th, a female (postmortem mantle length = 213 mm, body weight = 233.1 g) that appeared fully mature due to the large number of oocytes visible in its oviducts was removed from the tank and placed in a white, polystyrene, plastic (Styrofoam) box measuring 52 × 32 × 31 cm (L x W x H) and filled with seawater from the tank. The date that this squid had been put in the tank was not known, but she had remained in the tank between 3 and 24 days. After the squid was introduced into the plastic box, the temperature was increased gradually to 20.3 °C by adding 22.3 °C seawater. The final volume of seawater in the box was *ca*. 45 liters. Captive experiments suggest *T. pacificus* females ovulate and spawn at 19-23 °C^24^, so this 5-6 °C increase in temperature was used as a thermal stimulant to induce spawning. A smaller glass tank was placed inside the box to maneuver the squid above a waterproof digital videocamera (DMC-FT5, Panasonic Corp., Japan) placed on the bottom of the box pointed upward.

All procedures and experiments using squid were carried out in accordance with the “*Guidelines for the Care and Welfare of Cephalopods in Research*”^25^ and with the experimental guidelines set by Hokkaido University^26^.

## Supplementary Information

Supplementary Video 1

Supplementary Video 2

Supplementary Information

## Data Availability

All data generated or analyzed during this study are included in this published article and its Supplementary Information Files (Video 1 and Video 2).
