# Supplementary material for: Close-up observations on the spawning behavior of a captive Japanese flying squid (Todarodes pacificus)
Source: Sci Rep. 2019 Dec 24;9:19739. doi: 10.1038/s41598-019-56071-0 (PMC6930201; doi:10.1038/s41598-019-56071-0)
Supplement: Supplementary file 3 — Supplementary Information [file 41598_2019_56071_MOESM3_ESM.docx]

**Supplementary information**

Video 1. Spawning behavior of a Japanese flying squid – before ejection of oocytes. Key stages (including time (hr:min:sec) after transfer to box) were as follows: mantle-contraction rate increases rapidly (01:04:20); mantle and arms convulse briefly and body whitens (01:04:24); jelly slowly extrudes through funnel arms, and protective membrane spreads (01:04:27); jelly passes between arms IV toward mouth (01:04:36). Elapsed time after transfer to box is shown in the upper left.

Video 2. Spawning behavior of a Japanese flying squid – ejection of oocytes and release of translucent filaments (presumably spermatozoa). Key stages (including time (hr:min:sec) after transfer to box) were as follows: jelly reaches near tips of dorsal arms (arms I) (about 01:05:50); ejection of oocytes begins (1:50:55); ejection of oocytes becomes more rapid (about 01:06:00). Ejection of strands (presumably sperm) (1:06:01) begins. Elapsed time after transfer to box is shown in the upper left.
